# Effect of a Culturally Based Intervention Model on Infant Weight and Maternal Perceptions of Breastfeeding Adequacy Following Caesarean Section: Quasi-Experimental Study

**DOI:** 10.2196/75203

**Published:** 2026-06-22

**Authors:** Tri Budiati, Setyowati Setyowati, J M Seno Adjie, Jajang Gunawijaya

**Affiliations:** 1 Faculty of Nursing, Universitas Indonesia, Kampus FIK UI Depok, Jl Prof. Dr. Bahder Djohan, Depok Jawa Barat, Depok, 16424, Indonesia, 62 81290335195

**Keywords:** baby weight, cesarean section, culturally based intervention, perception of breastfeeding

## Abstract

**Background:**

The breastfeeding coverage rate (that is, the number of mothers reached by breastfeeding programs) in several regions in Indonesia, including Banten and West Java, is still below the actual rate of breastfeeding nationwide. The breastfeeding coverage rate is influenced by mothers’ condition after childbirth, particularly among mothers who have had a caesarean section. Support from partners, family, and close friends also has a large influence on the continuation of exclusive breastfeeding.

**Objective:**

This study aims to identify the effectiveness of a culturally based intervention and its effect on mothers’ perceptions of breast milk adequacy and the weight of newborns after caesarean section.

**Methods:**

This study used a quasi-experimental design involving 116 respondents who were grouped into control and intervention groups. Pre- and postintervention measurements were taken using the Perceived Insufficient Milk scale (*r*=0.976) and calibrated baby weight scales. Paired *t* tests were used for the analysis.

**Results:**

There was a significant increase in perception of breast milk adequacy and newborn weight in the intervention group compared to the control group. The intervention also increased the odds of perceiving breastfeeding as “very sufficient” by 2.53 times (odds ratio 2.53, 95% CI 1.36-4.73) after controlling for culturally held myths regarding breast milk insufficiency and family support.

**Conclusions:**

This model could be applied as an alternative intervention by health care personnel, especially nurses, to support postpartum mothers who have undergone a cesarean section.

## Introduction

Caesarean birth is one of the most-chosen delivery methods due to its advanced technology. A recent study reported that the proportion of caesarean births increased from 16.72% to 21.50% from 1998 to 2021, and it is projected to increase to nearly 29% by 2030 [1]. Maternal requests for caesarean section are associated with beliefs that recovery is painless and that it is the safest way to give birth, and these requests are also associated with traumatic previous birth experiences [2]. At the regional level, Asia has had the highest increase in recent years, with an average yearly increase of 6.4%. This increase is related to health care facility capacity, availability of resources, clinical management protocols, area of residence, maternal education, and economic status [1,3,4]. In Indonesia the increase in caesarean sections was approximately 10% from 2007 to 2017 [3]. This is due to an increasing number of pregnancy complications necessitating planned termination of pregnancy as well as increasingly advanced technology [4]. Unskilled health care workers, a lack of proper health infrastructure, and insufficient obstetric care are related to the increase in the decision to undergo a caesarean section. Sometimes, there are concerns related to vaginal appearance after vaginal birth, that is, that it will loosen the labia and decrease spousal satisfaction during sex; to minimalize this effect, mothers choose caesarean delivery [5]. Caesarean delivery increases the use of formula feeding in the hospital and delays the early initiation of breastfeeding. A previous study showed that after caesarean delivery, babies had a low breastfeeding rate, especially at 6 months post partum, due to lack of milk production, physical discomfort, and lack of support from the family [6,7]. Mothers undergo caesarean sections under general anesthesia, which delays the initiation of breastfeeding. Moreover, prelacteal feeding is reported as being associated with suboptimal initial breastfeeding [8]. Previous research has also found that mothers who undergo caesarean sections are less informed regarding the benefits of breastfeeding than mothers who give birth vaginally [7].

In Indonesia, exclusive breastfeeding remains a public health challenge, including in West Java and Banten. Recent evidence from West Java reported that only 58.3% of mothers practiced exclusive breastfeeding, with early initiation of breastfeeding achieved by 57.8% of mothers [9]. Maternal knowledge, education, self-efficacy, and early initiation of breastfeeding were identified as key determinants of exclusive breastfeeding. In Lebak, Banten, breastfeeding-related beliefs and cultural practices remain prevalent, with 60% of respondents demonstrating such beliefs [10]. Together, these findings suggest that breastfeeding outcomes in these regions are influenced not only by biological factors but also by maternal perceptions, cultural beliefs, and early postpartum experiences. Such challenges may be more pronounced among post-caesarean mothers, who are at higher risk of delayed breastfeeding initiation and perceived breast milk insufficiency, underscoring the need for targeted and culturally based interventions.

Culture is recognized as a key component in breastfeeding, but it is relatively underexplored, with considerable variation among ethnic groups [11]. There is little literature providing guidance on which cultural components need to be identified and considered in the design and development of interventions. Independent variables related to social and cultural significance must be explored to identify differences in cultural elements, such as values and behaviors. Dodgson et al [12] found 4 patterns of influence on breastfeeding behavior in society: local culture and customs, messages received by the mother, life experiences, and social support. The influence of culture on breastfeeding determines whether programs will be successful in specific areas or ineffective in others [13,14].

Another factor influencing breastfeeding and the sustainability of exclusive breastfeeding is support from people in the mother’s immediate environment. Previous research conducted by Budiati et al [15] found that the habits of and recommendations from close family members, as well as a lack of partner support, influenced exclusive breastfeeding among post-caesarean mothers in the Cibinong area of West Java. This study aims to identify the experience of husbands and family members in navigating the culture surrounding exclusive breastfeeding in post-caesarean mothers.

## Methods

### Overview

This was a quasi-experimental study with 116 respondents who were grouped into control and intervention groups. Respondents were recruited using consecutive sampling at 2 locations: Lebak, Banten, and Cimanggis, Bogor. Eligible participants were postpartum mothers starting from the seventh day after delivery. Four questionnaires were used to evaluate the effectiveness of the intervention through quantitative analysis: questionnaire A covered respondent data and characteristics; questionnaire B covered breastfeeding and infant weight data on the first day and on the seventh day after the intervention (assessment of feeding practices used a 24-hour recall question); questionnaire C evaluated maternal knowledge of exclusive breastfeeding practices (Cronbach α=0.801); and questionnaire D, which has been previously used and validated by Budiati et al [15] (validity coefficient=0.976), assessed the perception of the adequacy of breastfeeding (Multimedia Appendix 1). The Perceived Insufficient Milk (PIM) instrument [16] and calibrated baby weight scales were also used.

The culturally based intervention was developed during the model development stage based on an initial analysis and literature review. A culturally sensitive intervention module was created as a reference for post-caesarean mothers, husbands, families, and local health care volunteers. The intervention was implemented through training and assistance for mothers and families to support breastfeeding practices in a culturally appropriate manner. The modules covered breastfeeding benefits and content, breast care, proper attachment, lactation management, family empowerment and support, the role of health workers, and the involvement of community, traditional, and religious leaders. The intervention aimed to improve breastfeeding practices and breast milk adequacy, reflected in infant weight gain.

Data were analyzed using the Kolmogorov-Smirnov test, unpaired and paired *t* tests, and logistic regression.

### Ethical Considerations

Ethical approval for this research was obtained from the Faculty of Nursing Ethics Committee at the University of Indonesia (296/UN2.F12.D/HKP.02.04/2018). All participants provided written informed consent prior to enrollment. Participants were not offered any compensation for their participation in this study.

### Model Development

The culturally based intervention model was developed based on a stage 1 analysis and literature study. This model then became a reference for mothers, husbands, their families, and local health care volunteers. Training and assistance were provided to the mothers and families in implementing culturally sensitive care after caesarean delivery to promote breastfeeding practices, which we expected would result in increased breast milk production and adequate infant weight.

### Modules

**Figure 1.**
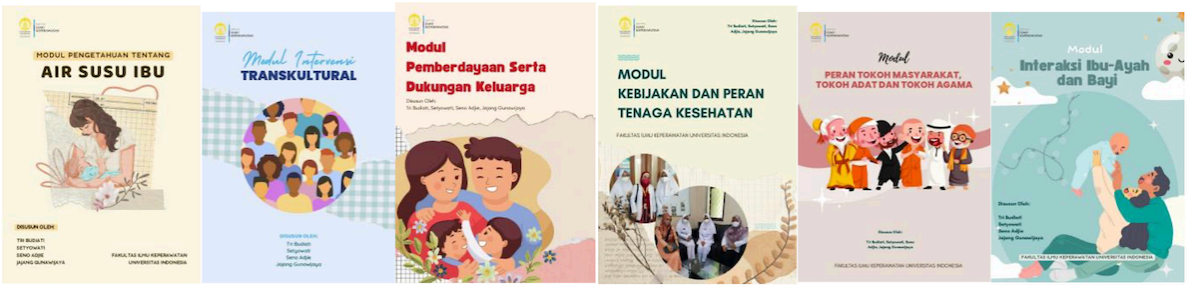
Modules developed for the culturally based breastfeeding intervention for post-caesarean mothers.

The modules shown in Figure 1 were developed during the stage 1 analysis to explain the benefits of breast milk, breast care during breastfeeding, proper attachment during breastfeeding, and breastfeeding management. Other modules addressed transcultural care and the role of the community in supporting post-caesarean mothers.

### Eligibility Criteria

Participants were included if they were post-caesarean mothers who had given birth to a full-term baby with a weight ≥2500 grams and an Apgar score ≥8, were living with their spouse at the same residence, and were able to write and communicate verbally and non-verbally. Mothers with a critical medical condition that required emergency intervention and who could not fully participate in the research process were excluded.

### Intervention

The culturally based intervention was carried out in 3 sessions of 60 minutes each. Training and assistance were scheduled 3 times for local health care volunteers, 3 times for family members, and 2 times for professional health care workers. A pretest was conducted at the first intervention session and repeated at the final session as a posttest to evaluate intervention outcomes. Meanwhile, the control group received conventional postpartum care delivery by health care professionals following the national maternal and child health reference (Buku Kesehatan Ibu dan Anak/KIA booklet) without any additional cultural components.

## Results

The results of this study include the respondents’ characteristics, breastfeeding outcomes, and factors associated with breastfeeding adequacy.

Table 1 shows that most respondents had a secondary education level and low family income. The control and intervention groups were generally homogeneous, except for family income (*P*=.02). Common myths related to breastfeeding identified in this study included beliefs that colostrum is harmful and should be discarded, that breast milk alone is insufficient to satisfy a newborn, and that caesarean delivery permanently impairs milk production. Family members frequently reinforced these beliefs by encouraging early formula supplementation when the infant appeared fussy or when the mother expressed uncertainty about her milk supply.

As shown in Table 2, the median maternal age and breastfeeding knowledge scores were comparable between the intervention and control groups, indicating homogeneity between groups (*P*>.05).

**Table 1.** Distribution of respondent characteristics (n=116).

Variable		Intervention group, n (%)	Control group, n (%)	Total, n (%)	*P* value
Education level					.78
	High	19 (32.7)	16 (27.6)	35 (30.2)	
	Middle	32 (55.2)	32 (55.2)	64 (55.1)	
	Basic	7 (12.1)	10 (17.2)	17 (14.7)	
	Total	58 (100.0)	58 (100.0)	116 (100.0)	
Family income					.02
	High	27 (46.6)	19 (32.8)	46 (39.7)	
	Low	31 (53.4)	39 (67.2)	70 (60.3)	
	Total	58 (100.0)	58 (100.0)	116 (100.0)	
Breastfeeding experience					.83
	Ever experienced	26 (44.8)	33 (56.9)	59 (50.9)	
	Never experienced	32 (55.2)	25 (43.1)	57 (49.1)	
	Total	58 (100.0)	58 (100.0)	116 (100.0)	
Culturally held myths					.62
	Supported	22 (37.8)	26 (56.9)	48 (41.3)	
	Not supported	36 (55.2)	32 (43.1)	68 (58.7)	
	Total	58 (100.0)	58 (100.0)	116 (100.0)	
Family support					.45
	Good	18 (31.1)	22 (37.8)	40 (34.5)	
	Bad	40 (68.9.)	36 (55.2)	76 (65.5)	
	Total	58 (100.0)	58 (100.0)	116 (100.0)	
Skin to skin contact					.44
	Yes	0 (0)	0 (0)	0 (0)	
	No	58 (100.0)	58 (100.0)	116 (100.0)	
	Total	58 (100.0)	58 (100.0)	116 (100.0)	

**Table 2.** Distribution of respondent characteristics among the two groups (n=116).

Group		Median (IQR)	Range	95% CI	*P* value
Age (years)					.54
	Intervention (n=58)	26.50 (21-32)	17‐39	27.14‐29.78	
	Control (n=58)	30 (24-36)	18‐42	30.03‐33.26	
Knowledge (score)					.78
	Intervention (n=58)	84.27 (69.71-98.83)	41.76‐100	78.00‐85.66	
	Control (n=58)	85.50 (71.39-99.61)	43.56‐100	79.72‐85.26	

Table 3 shows the distribution of breast milk production in the intervention and control groups before and after the culturally based intervention. Before the intervention, there was no significant difference in breast milk production between the intervention and control groups (*P*=.57), indicating comparable baseline conditions. After the intervention, a significant difference in breast milk production was observed between the two groups (*P*<.001). In the intervention group, the proportion of mothers who perceived their breast milk as “very adequate” increased markedly, from 46.6% before the intervention to 82.8% after the intervention. In contrast, the control group decreased in the “very adequate” category (from 54.8% to 41.4%), with most respondents moving to the “adequate” category. These findings indicate that cultural based intervention had a significant effect on improving perceived breast milk production among post-caesarean mothers.

**Table 3.** Breast milk production and infant weight before and after the intervention (n=116).

Breast milk production	Intervention group, n (%)	Control group, n (%)	Total, n (%)	*P* value^a^
Before intervention				.57
Not adequate	9 (15.5)	10 (17.0)	19 (16.3)	
Adequate	22 (37.9)	16 (28.2)	38 (33.2)	
Very adequate	27 (46.6)	32 (54.8)	59 (48.5)	
Total	58 (100.0)	58 (100.0)	116 (100.0)	
After intervention				<.001
Not adequate	1 (1.7)	7 (0)	1 (0.9)	
Adequate	9 (15.5)	34 (58.6)	43 (37.1)	
Very adequate	48 (82.8)	24 (41.4)	72 (62.0)	
Total	58 (100.0)	58 (100.0)	116 (100.0)	

a Paired *t *test.

Table 4 presents a comparison of infant weight before and after the culturally based intervention in both the intervention and control groups. In the control group, there was no significant change in infant weight before and after the intervention period (*P*=.56). Conversely, in the intervention group, infant weight increased significantly after the intervention (*P*=.001). The mean increase in infant weight in the intervention group was 185.60 (SD 119.64) grams, which was significantly higher than the change observed in the control group. These results demonstrate that the culturally based intervention contributed to a significant improvement in infant weight gain during the early postnatal period.

**Table 4.** Change in infant weight (n=116).

		Weight (grams), mean (SD)	Weight (grams), mean difference (SD)^a^	95% CI	*P* value^b^
Control group			–17.32 (203.05)	(–36.54 to 67.20)	.56
	Before	3204.89 (385.03)			
	After	3187.57 (413.62)			
Intervention group			185.60 (119.64)	(–147.06 to –84.15)	.001
	Before	3256.63 (365.04)			
	After	3442.23 (369.07)			

a Independent *t* test.

b Kolmogorov Smirnov test.

There were significant improvements in perceived breast milk production and infant weight before and after the intervention in the intervention group (P=0.001 and P<0.001, respectively). In contrast, no significant changes were observed in the control group (P=0.56) . Moreover, a comparison of infant weight change between the intervention and control groups demonstrated a statistically significant difference (P=0.001) , indicating that infants in the intervention group experienced greater weight gain than those in the control group.

As shown in Table 5, family support, culturally held myths, income, and the culturally based intervention had *P* values <.25; these variables were thus used in multivariate modeling.

Table 6 shows that the strongest way to increase breast milk production and infant weight was the culturally based intervention (odds ratio 2.53; 95% CI 1.36-4.73) after controlling for belief in culturally held myths and family support.

**Table 5.** Bivariate analysis results.

Variable	*P* value
Age	.69
Family support	.08^a^
Breastfeeding experience	.36
Belief in culturally held myths	.08^a^
Education	.46
Work	.76
Income	.24^a^
Knowledge	.24
Culturally based intervention	<.001^a^

a *P* value <.25 (used in multivariate modeling).

**Table 6.** Logistic modeling.

Variable	B	SE	Wald	*df*	P value	Odds ratio (95% CI)
First model						
Income^a^	0.525	1.720	0.93	1	.76	1.691 (0.06‐49.22)
Breastfeeding knowledge^a^	0.420	1.584	0.70	1	.79	1.522 (0.07‐33.95)
Belief in culturally held myths	–1.395	0.351	15.806	1	.001	0.248 (0.13‐0.49)
Culturally based intervention	–1.375	0.339	16.494	1	<.001	0.253 (1.13‐0.49)
Family support	0.419	0.325	1.664	1	.20	1.521 (0.80‐2.86)
Constant	–0.511	1.384	0.136	1	.71	—^b^
Second model						
Culturally based intervention	0.928	0.319	8.450	1	.004	2.530 (1.36‐4.73)
Myth	0.722	0.509	2.015	1	.16	2.059 (0.76‐5.58)
Family support	0.751	0.516	2.114	1	.15	2.119 (0.77‐5.83)
Constant	–0.365	0.517	0.499	1	.48	0.694

a Income and breastfeeding knowledge were excluded from the second model following backward elimination due to *P* values exceeding the retention threshold (*P*>.25).

b Not applicable.

## Discussion

### Principal Findings

The majority of respondents in this study did not perform skin-to-skin contact, even though it is the first step in successful breastfeeding. This is consistent with research indicating that the caesarean process is associated with delays in both skin-to-skin contact and lactogenesis [7,8,17,18]. Disruption to the lactogenesis process can be influenced by the effects of decreased oxytocin secretion or stress in mothers who have undergone a caesarean section, resulting in decreased breast milk production in the mother [19,20].

According to Budiati and Setyowati [21], although mothers may have good knowledge, they still face many environmental obstacles to practicing the desired behavior. Also, many mothers may recognize the benefits of exclusive breastfeeding, but they are constrained by sociocultural barriers, such as a lack of family support, gender roles, traditional customs, and body image [22].

Our culturally based intervention combined several modules proven to affect breast milk adequacy and infant weight. This is in line with previous research on a baby-friendly hospital initiative that combined two or more strategies, finding that it was sustainable in different hospitals and in the community [23].

Indonesian culture is based on kinship, and mothers’ decisions about breastfeeding are often influenced by the people around them, such as their own mothers, parents-in-law, and husbands [22]. Thus, subjective norms are also a key component of successful breastfeeding [24,25]. Breastfeeding control refers to a mother’s self-confidence and ability to practice breastfeeding.

During the model development stage, preliminary qualitative findings revealed that mothers initially desired exclusive breastfeeding but felt helpless and deferred to family members who introduced formula milk when breast milk appeared insufficient or the infant was fussy. This is consistent with previous research that mentioned how social influence affects mothers’ decisions to breastfeed during the first 3 days after delivery [26]. That study showed that social norms or influence come from family, neighbors, and close relatives. These people’s beliefs can unintentionally influence the mother’s beliefs. If the family, neighbors, or close relatives feel that the mother should breastfeed exclusively, then she will do so. Conversely, mothers who receive opposing social pressure share the same perceptions as the people around them [27].

The results of this study showed that the average weight of infants in the intervention group differed before and after the culturally based intervention. This is in line with previous research regarding the effectiveness of breastfeeding education on infant weight and breastfeeding mothers’ self-efficacy [28,29]. Other research on low-birth-weight infants highlighted that health education combined with peer support could increase maternal confidence and infant weight through the implementation of “kangaroo mother” care (ie, increased skin-to-skin contact) and lead to increased breastfeeding [30].

The findings of this study highlight the important role of cultural and social factors in shaping exclusive breastfeeding practices. This is consistent with findings from Herawati et al [10], who reported that breastfeeding beliefs and cultural factors significantly influenced exclusive breastfeeding success in Banten and West Java. These results suggest that cultural influences on breastfeeding may vary across regions, depending on local beliefs, family structures, and social dynamics.

Furthermore, the results align with a global scoping review by Badanta et al [31], which demonstrated that cultural beliefs and social norms strongly affect women’s breastfeeding behaviors. Common cultural perceptions, such as beliefs regarding the quality of breast milk or the value of colostrum, as well as family and social pressures, were identified as barriers to exclusive breastfeeding. By integrating culturally based approaches into the intervention, this study supports the growing evidence that breastfeeding promotion strategies should not focus solely on health education but also address underlying cultural beliefs and social contexts.

### Limitations

A limitation of this study was that several participants dropped out due to health problems after being exposed to COVID-19. In addition, there were confounding factors, such as self-efficacy, nutrition, and the use of galactagogues by mothers, that were difficult to control and may have affected the adequacy of breast milk production.

### Implications

Providing a program that combines several strategies to promote breastfeeding among mothers can increase exclusive breastfeeding, especially among mothers who have undergone a caesarean delivery. Our culturally based intervention could be applied in health care facilities, such as community health centers (*puskesmas*) and hospitals, as a first-line strategy in the care of pregnant women. In addition to medical practices, care and cultural interventions are factors that affect mothers’ decisions in choosing a delivery method. Culturally based interventions are often overlooked by health care workers, and developing new guidance and policies will increase mothers’ awareness of exclusive breastfeeding practices, especially among post-caesarean mothers.

### Conclusion

This quasi-experimental study was designed to evaluate the effectiveness of a culturally based intervention to increase breast milk adequacy and infant weight. The intervention increased the odds of perceiving breastfeeding as “very sufficient” by 2.53 times (odds ratio 2.53, 95% CI 1.36-4.73), after controlling for belief in breastfeeding myths and family support, compared to mothers who did not receive the intervention. The intervention improved mothers’ and family members’ capacity to differentiate evidence-based breastfeeding practices from culturally held myths, resulting in more informed and supportive family engagement during the post-caesarean recovery period.

## Supplementary material

10.2196/75203 Multimedia Appendix 1 Questionnaires created by the authors and used in the study.

## References

[1] Pandey AK, Raushan MR, Gautam D, Neogi SB (2023). Alarming trends of cesarean section-time to rethink: evidence from a large-scale cross-sectional sample survey in India. J Med Internet Res.

[2] Muhandule CJLS, Benetti CMS, Fogulin LB, Bento SF, Amaral E (2024). Caesarean delivery on maternal request: the perspective of the postpartum women. BMC Pregnancy Childbirth.

[3] Islam M, Shanto HH, Jabbar A, Howlader M (2022). Caesarean section in Indonesia: analysis of trends and socio.demographic correlates in three demographic and health surveys (2007-2017). Dr Sulaiman Al Habib Medical Journal.

[4] Kumar P, Srivastava S, Chaudhary P, Muhammad T (2023). Factors contributing to socio-economic inequality in utilization of caesarean section delivery among women in Indonesia: evidence from demographic and health survey. PLoS ONE.

[5] Wijaya PE, Fisher J, Kirkman M (2024). Female genital cosmetic surgery in Indonesia: a qualitative analysis of medical advertising on Instagram. Cult Health Sex.

[6] Li L, Wan W, Zhu C (2021). Breastfeeding after a cesarean section: a literature review. Midwifery.

[7] Ulfa Y, Maruyama N, Igarashi Y, Horiuchi S (2023). Early initiation of breastfeeding up to six months among mothers after cesarean section or vaginal birth: a scoping review. Heliyon.

[8] Vaz JS, Gatica-Domínguez G, Neves PAR, Vidaletti LP, Barros AJD (2022). Early initiation of breastfeeding is inversely associated with public and private c-sections in 73 lower- and middle-income countries. Sci Rep.

[9] Apriningsih, Hanifah L, Nasrulloh N (2024). Exclusive breastfeeding practice during COVID-19 pandemic in West Java Indonesia: a cross-sectional study. PLoS ONE.

[10] Herawati I, Maiselamah, Widiastuti R (2025). Evaluation of the influence of social and biological factors on the success of exclusive breastfeeding and increased baby weight in Jakarta, West Java and Banten. Jurnal Penelitian Pendidikan IPA.

[11] Gutierrez-de-Terán-Moreno G, Ruiz-Litago F, Ariz U (2022). Successful breastfeeding among women with intention to breastfeed: from physiology to socio-cultural factors. Early Hum Dev.

[12] Dodgson JE, Tarrant M, Fong DYT, Peng XH, Hui WHC (2003). Breastfeeding patterns of primiparous mothers in Hong Kong. Birth.

[13] Vilar-Compte M, Pérez-Escamilla R, Ruano AL (2022). Interventions and policy approaches to promote equity in breastfeeding. Int J Equity Health.

[14] Hidrobo-Guzmán JF, Morejón-Jácome GE, Cárdenas-Robles ED, Pilco-Vargas LD, Posso López DV, Iguago Angamarca ET (2025). Effectiveness of ethnic-cultural educational strategies for the promotion of breastfeeding. Int J Environ Res Public Health.

[15] Budiati T, Setyowati S, Helena N (2010). Peningkatan produksi ASI ibu nifas seksio sesarea melalui pemberian paket “Sukses ASI”. Jurnal Keperawatan Indonesia.

[16] McCarter-Spaulding DE, Kearney MH (2001). Parenting self-efficacy and perception of insufficient breast milk. J Obstet Gynecol Neonatal Nurs.

[17] Juan J, Zhang X, Wang X (2022). Association between skin-to-skin contact duration after caesarean section and breastfeeding outcomes. Children (Basel).

[18] Lian W, Ding J, Xiong T, Liuding J, Nie LT (2022). Determinants of delayed onset of lactogenesis II among women who delivered via cesarean section at a tertiary hospital in China: a prospective cohort study. Int Breastfeed J.

[19] Li S, Wupuer T, Hou R (2024). Factors influencing delayed onset of lactogenesis: a scoping review. Int J Gen Med.

[20] Kraus V, Čižmárová B, Birková A (2025). When your body tells you to not breastfeed-the connivance of oxytocin, prolactin, and dopamine. Int J Mol Sci.

[21] Budiati T, Setyowati (2019). The influence culture and maternal care on exclusive breastfeeding practice in post caesarean section mothers. Enfermería Clínica.

[22] Budiati T, Adjie S, Gunawijaya J, Setyowati S (2021). Fathers’ role in sustainability of exclusive breastfeeding practice in post-cesarean-section mothers. J Public Health Res.

[23] Kim SK, Park S, Oh J, Kim J, Ahn S (2018). Interventions promoting exclusive breastfeeding up to six months after birth: a systematic review and meta-analysis of randomized controlled trials. Int J Nurs Stud.

[24] Wu L, Li X, Ismail HN, Guo P, Yang J (2025). Understanding the psychosocial dilemma in breastfeeding: a narrative review of extended theory of planned behavior and its intervention strategies. Reprod Health.

[25] Bartle NC, Harvey K (2017). Explaining infant feeding: the role of previous personal and vicarious experience on attitudes, subjective norms, self-efficacy, and breastfeeding outcomes. Br J Health Psychol.

[26] Hobbs AJ, Mannion CA, McDonald SW, Brockway M, Tough SC (2016). The impact of caesarean section on breastfeeding initiation, duration and difficulties in the first four months postpartum. BMC Pregnancy Childbirth.

[27] Santacruz-Salas E, Aranda-Reneo I, Segura-Fragoso A, Cobo-Cuenca AI, Laredo-Aguilera JA, Carmona-Torres JM (2019). Mothers’ expectations and factors influencing exclusive breastfeeding during the first 6 months. Int J Environ Res Public Health.

[28] Balogun OO, Dagvadorj A, Anigo KM, Ota E, Sasaki S (2015). Factors influencing breastfeeding exclusivity during the first 6 months of life in developing countries: a quantitative and qualitative systematic review. Matern Child Nutr.

[29] Güneş AO, Karadağ N, Karatekin G (2023). The effect of breastfeeding self-efficacy on infants’ weights and breastfeeding outcomes. Turk Arch Pediatr.

[30] Rustina Y, Budiati T (2019). Peer support increases maternal confidence, kangaroo mother care implementation and weight gain in LBW infants. Compr Child Adolesc Nurs.

[31] Badanta B, Suarez-Reina P, Álvarez-Pérez I (2025). Cultural beliefs and practices about women’s breastfeeding behaviors: a scoping review. Enf Global.

