# Supplementary material for: Effect of a Culturally Based Intervention Model on Infant Weight and Maternal Perceptions of Breastfeeding Adequacy Following Caesarean Section: Quasi-Experimental Study
Source: Asian Pac Isl Nurs J. 2026 Jun 22;10:e75203. doi: 10.2196/75203 (PMC13286324; doi:10.2196/75203)
Supplement: Multimedia Appendix 1 [file apinj-v10-e75203-s001.pdf]

QUESTIONNAIRE A

Respondent Demographic Data Questionnaire

Instructions

Please answer each question honestly in the space provided.

- Name :
- WhatsApp/Phone Number :
- Mother's Age :
- Infant's Age :
- Current Infant Weight :
- Education :
    - 0 = No formal education
    - 1 = Elementary School
    - 2 = Junior High School or equivalent
    - 3 = Senior High School or equivalent
    - 4 = Higher Education (Diploma/Bachelor/Master/Doctoral Degree)
- Occupation :
    - 0 = Unemployed
    - 1 = Employed
- Number of Children :
- Family Income :
  What is the total monthly income of the father and mother?
    - □ < IDR 2,773,500
    - □ ≥ IDR 2,773,500
- Ethnicity :

After birth, was the baby placed on the mother's chest?

- □ Yes
- □ No

If yes, for how long?

- □ < 60 minutes
- □ ≥ 60 minutes

QUESTIONNAIRE B

Exclusive Breastfeeding Success Questionnaire

1. Knowledge

Instructions

Please mark the appropriate answer according to your opinion.

No	Statement	True	False
1	Exclusive breastfeeding is the best nutrition for infants aged 0–6 months		
2	Exclusive breastfeeding is suitable for infants because it is easily digested		
3	Exclusive breastfeeding should only be given until the infant is 4 months old		
4	Exclusive breastfeeding contains good nutrients for infant growth		
5	Exclusive breastfeeding causes allergies in infants		
6	Exclusive breastfeeding strengthens the emotional bond between mother and baby		
7	Exclusive breastfeeding causes diarrhea in infants		
8	Exclusive breastfeeding helps infant development		

2. **Attitude**

Instructions

Please mark the answer that best reflects your opinion or condition.

Response Options:

SA= Strongly Agree

A=Agree

DA=Disagree

SD= Strongly Disagree

No	Statement	SD	DA	A	SA
9	I will provide complementary feeding before my baby reaches 6 months of age				
10	I will provide complementary feeding after my baby reaches 6 months old				
11	I will continue breastfeeding even after my baby is older than 6 months				
12	I will replace exclusive breastfeeding with porridge if my baby is allergic to breast milk				
13	I will inform other mothers if they do not know about exclusive breastfeeding				
14	If my baby continues crying during exclusive breastfeeding, I will provide bananas, formula milk, or other supplementary foods				
15	I will introduce complementary foods as early as possible				
16	If my baby dislikes exclusive breastfeeding, I will only provide supplementary foods				

3. **Practice/Behavior**

Instructions

Please mark the answer according to your practice or condition.

Response Options:

N = Never

R = Rarely

O = Often

A = Always

No	Statement	N	R	O	A
17	I provide only exclusive breastfeeding until my baby is 6 months old				
18	I provide exclusive breastfeeding combined with porridge, mashed bananas, or formula milk before 6 months				
19	I first provide complementary foods after my baby is 6 months old				
20	I discard the first breast milk (colostrum/yellowish milk)				
21	I prioritize exclusive breastfeeding over formula milk				
22	I provide formula milk since birth				
23	I provide supplementary foods when my breast milk supply is insufficient				
24	I provide formula milk more frequently than exclusive breastfeeding				

25 What challenges have you experienced during exclusive breastfeeding?…………

Maternal Satisfaction with Breast Milk Production Questionnaire

Instructions

Mothers are expected to answer all statements below.

Put a check mark (√) in the appropriate column.

Response Options:

1 = Strongly Disagree

2 = Disagree

3 = Agree

4 = Strongly Agree

No	Statement	1	2	3	4
1	I feel happy because I was able to breastfeed my baby early				
2	I feel happy because my baby becomes healthier by consuming only breast milk				
3	I can rest well because my baby is not fussy				
4	I feel happy because my breast milk is sufficient for my baby				
5	I feel happy because my breast milk production is smooth				
6	I feel happy because I do not need to buy formula milk				

Observation Sheet (Completed by Data Collector)

Breast Milk Production of Breastfeeding Mothers

No	Observed Aspect	Yes	No
1	Mother's breasts appear tense/full		
2	Baby appears calm		
3	Let-down reflex functions well		
4	Baby sucks strongly with a slow rhythm		
5	Baby breastfeeds calmly		
6	Mother does not appear to feel pain		
7	Mother expresses milk because breasts are full		
8	Baby is given formula milk		
9	Mother uses both breasts alternately and baby breastfeeds until satisfied		
10	After breastfeeding, baby falls asleep and releases the breast spontaneously		
11	Mother breastfeeds on demand		
12	Mother appears relaxed		
13	Nipples and areola are clean and not cracked		
14	Mother's breasts appear empty after breastfeeding		
15	Mother can provide expressed breast milk using a cup and spoon		

QUESTIONNAIRE C
Exclusive Breastfeeding Practice Questionnaire

Instructions
Please place a check mark according to your condition.

No	Statement	Yes	No
1	Breastfeeding may cause breasts to sag; therefore young mothers should avoid breastfeeding		
2	Breastfeeding strengthens the bond between mother and baby		
3	Infants aged 0–6 months should receive exclusive breastfeeding to stay healthy		
4	Formula feeding is considered better and more modern than exclusive breastfeeding		
5	Colostrum is spoiled milk and should not be given to the baby		
6	Colostrum may cause stomachache and diarrhea in infants		
7	Breast milk produced in the first days after birth is insufficient for the baby		
8	Breast milk produced by mothers is destined by God to be sufficient for infants		
9	If breast milk has not come out on the first day after delivery, babies may be given sugar water, coffee, formula milk, or other drinks		
10	Formula feeding in newborns may cause nipple confusion/refusal to breastfeed		
11	If the baby refuses to suck, nipples should be smeared with honey so the baby will breastfeed		
12	Infants younger than 6 months may be given bananas, dates, or porridge to make them fuller and healthier		
13	Combining formula milk and breast milk is considered very beneficial even before 6 months		
14	Exclusive breastfeeding alone is sufficient for infant nutritional needs during the first 6 months		
15	Newborns should be given honey/rice water/coconut water/other drinks to facilitate digestion or meconium passage		

Please explain other breastfeeding practices (foods/drinks given to infants under 6 months not listed above) and the reasons.

If none, please put a dash (-).

………………………………………………………………………………..

QUESTIONNAIRE D
FAMILY SUPPORT FOR EXCLUSIVE BREASTFEEDING QUESTIONNAIRE

Instructions

Please recall the support provided by your family during the time you breastfed your baby aged 0–6 months. Put a check mark according to your experience.

Response Options:

N= Never

R= Rarely

O= Often

A= Always

No	Statement	N	R	O	A
1	Family members informed me that infants aged 0–6 months should receive only breast milk without additional food or drinks				
2	Family members informed me about storing expressed breast milk in the refrigerator/freezer				
3	Family members helped me find information about exclusive breastfeeding				
4	Family members prohibited sharing information about exclusive breastfeeding				
5	Family members encouraged giving complementary foods or formula milk during exclusive breastfeeding				
6	Family members allowed me to remain uninformed about exclusive breastfeeding practices				

Instrumental Support

No	Statement	N	R	O	A
7	Family members provided nutritious food during exclusive breastfeeding				
8	Family members accompanied me to breastfeeding				

No	Statement	N	R	O	A
	education sessions				
9	Family members bought breast pumps, storage bags, bottles, or cups for expressed milk				
10	Family members refused to help with household chores while I was breastfeeding				
11	Family members bought formula milk during exclusive breastfeeding				
12	Family members did not help provide expressed breast milk when I was working				

Emotional Support

No	Statement	N	R	O	A
13	Family members asked about difficulties I experienced during exclusive breastfeeding				
14	Family members accepted my condition during exclusive breastfeeding				
15	Family members encouraged and motivated me to provide exclusive breastfeeding				
16	Family members listened to my complaints during exclusive breastfeeding				
17	Family members showed little sympathy toward my breastfeeding difficulties				
18	Family members refused to help when I needed assistance during breastfeeding				
19	Family members scolded me for exclusively breastfeeding my baby				

Appraisal Support

No	Statement	N	R	O	A
20	Family members praised me for providing exclusive breastfeeding				
21	Family members trusted my ability to provide exclusive breastfeeding				
22	Family members appreciated my efforts in exclusive breastfeeding				
23	Family members allowed me to provide formula milk or complementary feeding before 6 months				
24	Family members praised me for giving complementary foods during exclusive breastfeeding				
25	Family members doubted my ability to provide exclusive breastfeeding				

Sources of Family Support

Which family member played the most important role in supporting exclusive breastfeeding?

1 = Husband

2 = Biological Mother

3 = Mother-in-law

4 = Other relatives (siblings/in-laws/cousins)

Which family member did not support exclusive breastfeeding?

1 = Husband

2 = Biological Mother

3 = Mother-in-law

4 = Other relatives (siblings/in-laws/cousins)
